# Acromegaly at diagnosis in 3173 patients from the Liège Acromegaly Survey (LAS) Database

**DOI:** 10.1530/ERC-17-0253

**Published:** 2017-07-21

**Authors:** Patrick Petrossians, Adrian F Daly, Emil Natchev, Luigi Maione, Karin Blijdorp, Mona Sahnoun-Fathallah, Renata Auriemma, Alpha M Diallo, Anna-Lena Hulting, Diego Ferone, Vaclav Hana, Silvia Filipponi, Caroline Sievers, Claudia Nogueira, Carmen Fajardo-Montañana, Davide Carvalho, Vaclav Hana, Günter K Stalla, Marie-Lise Jaffrain-Réa, Brigitte Delemer, Annamaria Colao, Thierry Brue, Sebastian J C M M Neggers, Sabina Zacharieva, Philippe Chanson, Albert Beckers

**Affiliations:** 1 Department of EndocrinologyCHU de Liège, University of Liège, Belgium; 2 Clinical Centre of Endocrinology and GerontologyMedical University, Sofia, Bulgaria; 3 APHP Endocrinology and Reproductive DiseasesParis Sud University, Le Kremlin-Bicêtre, France; 4 Section of EndocrinologyDepartment of Medicine, Erasmus University Medical Center, Rotterdam, The Netherlands; 5 Department of EndocrinologyCentre de Référence des Maladies Rares d’Origine Hypophysaire, Hôpital de la Timone, Marseille, France; 6 Dipartimento Di Medicina Clinica e ChirurgiaSezione di Endocrinologia, University “Federico II”, Naples, Italy; 7 Department of EndocrinologyCHU de Reims, France; 8 Department of Molecular Medicine and SurgeryKarolinska University Hospital, Stockholm, Sweden; 9 Department of Internal MedicineUniversity of Genoa, Genova, Italy; 10 Third Department of Internal Medicine1st Faculty of Medicine, Charles University, Prague, Czech Republic; 11 Department of Biotechnological and Applied Clinical SciencesUniversity of L’Aquila, L’Aquila, Italy and Neuromed, IRCCS, Pozzilli, Italy; 12 Department of Internal MedicineEndocrinology and Clinical Chemistry, Max Planck Institute of Psychiatry, Munich, Germany; 13 Department of Internal MedicineEndocrinology, Diabetes and Metabolism Unit, Centro Hospitalar de Trás-os-Montes e Alto Douro, Portugal; 14 Department of EndocrinologyHospital Universitario de la Ribera, Alzira, Spain; 15 Department of EndocrinologyDiabetes and Metabolism, Centro Hospitalar S. João, Faculty of Medicine, Instituto de Investigação e Inovação em Saúde, University of Porto, Porto, Portugal

**Keywords:** acromegaly, comorbidity, database, data mining, diagnosis, growth hormone, IGF-1, pituitary adenoma, symptoms

## Abstract

Acromegaly is a rare disorder caused by chronic growth hormone (GH) hypersecretion. While diagnostic and therapeutic methods have advanced, little information exists on trends in acromegaly characteristics over time. The *Liège Acromegaly Survey (LAS) Database*, a relational database, is designed to assess the profile of acromegaly patients at diagnosis and during long-term follow-up at multiple treatment centers. The following results were obtained at diagnosis. The study population consisted of 3173 acromegaly patients from ten countries; 54.5% were female. Males were significantly younger at diagnosis than females (43.5 vs 46.4 years; *P* < 0.001). The median delay from first symptoms to diagnosis was 2 years longer in females (*P* = 0.015). Ages at diagnosis and first symptoms increased significantly over time (*P* < 0.001). Tumors were larger in males than females (*P* < 0.001); tumor size and invasion were inversely related to patient age (*P* < 0.001). Random GH at diagnosis correlated with nadir GH levels during OGTT (*P* < 0.001). GH was inversely related to age in both sexes (*P* < 0.001). Diabetes mellitus was present in 27.5%, hypertension in 28.8%, sleep apnea syndrome in 25.5% and cardiac hypertrophy in 15.5%. Serious cardiovascular outcomes like stroke, heart failure and myocardial infarction were present in <5% at diagnosis. Erythrocyte levels were increased and correlated with IGF-1 values. Thyroid nodules were frequent (34.0%); 820 patients had colonoscopy at diagnosis and 13% had polyps. Osteoporosis was present at diagnosis in 12.3% and 0.6–4.4% had experienced a fracture. In conclusion, this study of >3100 patients is the largest international acromegaly database and shows clinically relevant trends in the characteristics of acromegaly at diagnosis.

## Introduction

Acromegaly is caused by chronic hypersecretion of growth hormone (GH) and insulin-like growth factor-1 (IGF-1), usually due to a GH-secreting pituitary adenoma (somatotropinoma) (Melmed 2017). Acromegaly is a rare disorder; modern epidemiological data from various population-based (Daly *et al.* 2006, Fernandez *et al.* 2010) and insurance database studies (Burton *et al.* 2016) are available and suggest that acromegaly has a prevalence of 2.8–13.7 cases/100,000 and an incidence of 0.2–1.1 cases/100,000 (Lavrentaki *et al.* 2016).

Chronically elevated GH and insulin-like growth factor-1 (IGF-1) levels lead to a complex spectrum of signs that include acral overgrowth, facial changes, musculoskeletal disease or gigantism if the GH hypersecretion occurs before epiphyses have fused (Melmed 2017). Patients with active acromegaly also suffer from cardiovascular and metabolic abnormalities, including hypertension, arrhythmia, cardiomegaly, diabetes mellitus and dyslipidemia (Melmed *et al.* 2013). Together these lead to increased morbidity and mortality in acromegaly, predominantly due to cardiovascular and respiratory disease (Stewart & Sherlock 2012, Ritvonen *et al.* 2015, Ramos-Leví & Marazuela 2017). Bringing GH/IGF-1 levels within the normal range returns mortality to that of the general population, although the precise threshold at which risk normalization occurs is debated (Holdaway *et al.* 2008, Sherlock *et al.* 2010).

Methods for the management of acromegaly have evolved over the past 40 years and for most approaches, the efficacy and safety profiles are well documented. Neurosurgical techniques have been refined from the first trans-sphenoidal operations to new endoscopic techniques, while medical therapies now involve a range of options from somatostatin analogs (SSA) and somatostatin receptor ligands (SRL) to the growth hormone (GH) receptor antagonist pegvisomant and dopamine agonists (Melmed 2016). Radiotherapy techniques have undergone significant developments leading to the gamma-knife used today. Modern acromegaly therapy is guided by recommendations from consensus publications, with primary neurosurgery potentially offering cure in pituitary tumors that are smaller or uncomplicated (Giustina *et al.* 2010, Katznelson *et al.* 2014). In many patients, multimodal therapy is needed, particularly for those with aggressive disease or non-resectable tumors.

As a rare disease, studies on acromegaly have generally focused on relatively small populations or have addressed regional or national cohorts and patients enrolled in treatment-specific safety databases (Jenkins *et al.* 1995, Sherlock *et al.* 2009, Trainer 2009, Tritos *et al.* 2014). Data from such studies have provided valuable information about acromegaly and have contributed to improvements in patient management. Large international studies of the clinical characteristics and therapeutic evolution of unselected groups of acromegaly patients do not exist. We were interested in studying multiple aspects of acromegaly, including detailed assessments of large numbers of data points covering hormonal, pathological, genetic, clinical and therapeutic measures and how these factors are interrelated. We developed and deployed a relational database, the *Liège Acromegaly Survey (LAS) Database*, for the analysis of data collected from large populations of patients with acromegaly. Following preliminary studies to validate the data collection and analysis potential of the *LAS Database* (Theodoropoulou *et al.* 2009, Petrossians *et al.* 2012, Franck *et al.* 2017), we report the first comprehensive study of 3173 acromegaly in patients from 14 participating centers across Europe.

## Methods

The study included patients with an established diagnosis of acromegaly at the 14 study centers across Belgium (Centre Hospitalier Universitaire de Liège), Bulgaria (Medical University, Sofia), Czech Republic (Charles University, Prague), France (Paris Sud University, Le Kremlin-Bicêtre; Hôpital de la Timone, Marseille, Centre Hospitalier Universitaire de Reims), Germany (Max Planck Institute of Psychiatry, Munich), Italy (Federico II University, Naples; University of Genoa, Genoa; University of L’Aquila; and Neuromed, Pozzilli), the Netherlands (Erasmus University Medical Center, Rotterdam), Portugal (Centro Hospitalar S. João, Porto), Spain (Hospital Universitario de la Ribera, Alzira) and Sweden (Karolinska University Hospital, Stockholm).

The *LAS Database* is a relational database that permits the analysis of comprehensive arrays of data covering laboratory values, dose adaptation of treatment and clinical evolution. The goal of the *LAS Database* was to design a framework to capture available data on >2000 acromegaly patients and to permit statistically robust analysis of clinically relevant topics. The database management system was kept separate from the data capture interface. The open source mySQL server (Oracle, USA) was used to store the data, while the data capture interface used locally at each participating center was programmed using the Delphi RAD system. The initial development and validation of the framework is described in Petrossians and coworkers (Petrossians *et al.* 2012).

The current study ran from 30 September, 2012, to 1 January, 2015, and data cutoff for this analysis was 1 October, 2016. All patients with a diagnosis of acromegaly at the participating centers up to 1 January 2015 were valid for inclusion. Those with valid demographic data and at least one post-diagnosis/baseline follow-up dataset were included in the statistical analysis. There was no upper or lower limit to the duration of follow-up, number of treatments or treatment adaptations, drug dose alterations or hormonal/clinical/radiological results recorded over time. Complete data on the 147 variables that were collected over the course of the patient’s clinical follow-up were to be entered; when an assessment had not been performed (e.g. cardiac ultrasound, colonoscopy, polysomnography), these individuals were not included in the statistical analysis for that particular parameter. Hormonal data have evolved over time due to refinements in assay methodologies, which can lead to inconsistencies when comparing values. The *LAS Database* accounted for changes in GH assay reference ranges by automatically converting values in ng/mL to µU/mL based on the date and reference used in the center at that time. For IGF-1, absolute measured values were encoded along with the upper limit of normal for age and sex based on the assay used at the center. Results were then expressed as percent of upper limit of the normal value (%ULN). Radiological data for the maximal tumor diameter were used to calculate the proportion of patients with micro (<10 mm) and macroadenomas (≥10 mm) on MRI scans at diagnosis. Nodular thyroid disease was considered present when a solitary thyroid nodule or a multinodular goiter was confirmed on ultrasound. Diabetes was considered as being present when a diagnosis had been made in the medical history of the patient and/or a recorded glucose value of ≥200 mg/dL was found at 120 min during a standard oral glucose tolerance test (OGTT). Genetic studies were not performed specifically over the course of this study and only information on familial diseases or previously established genetic diagnoses was collected.

The study was performed under a central Ethics Committee approval covering all centers from the Centre Hospitalier Universitaire de Liège, while each individual center complied with their individual local ethics requirements and procedures. Data were encoded locally using the *LAS Database* data capture interface and each patient entered was assigned an anonymous study identifier. Patient identifying information was never shared with the central database where information from participating centers was pooled for analysis.

### Statistics

To examine the evolution of factors over time the study population was divided by study center, gender and decade of diagnosis. Data were analyzed using the R software package (R Core Team 2015; http://www.R-project.org) and graphics were plotted using the Lattice package (Lattice, Sarkar D. New York (2008). For continuous variables, data were plotted and tested for normality. As none of the variables had a normal distribution, data were expressed as median and interquartile range (IQR) from the first to the third quartile (25th and 75th percentiles). Data distribution was represented graphically with density graphs using Gaussian kernel smoothing with individual data points plotted at the abscissa (‘rug’). Data spreads were drawn using boxplots showing the medians and interquartile ranges, while the whiskers represented 1.5 times the interquartile ranges. Statistical comparisons were performed using the Mann–Whitney test. Single and multiple regression analyses were performed using generalized additive models. Count variables were compared using the *χ*^2^ test. Time data were analyzed either continuously for regression models or divided into four groups (before 1990, 1990–1999, 2000–2009, 2010 and after). The earliest date (pre/post 1990) was chosen as it represents a period when new diagnostic (MRI) and therapeutic (somatostatin analogs) modalities were becoming generally available. Patient ages were also analyzed either as continuous values for regression and Mann–Whitney tests or grouped into categories: 0–29 years, 30–49 years, 50–64 years and ≥65 years.

## Results

### Study population and demographics

**Figure 1:**
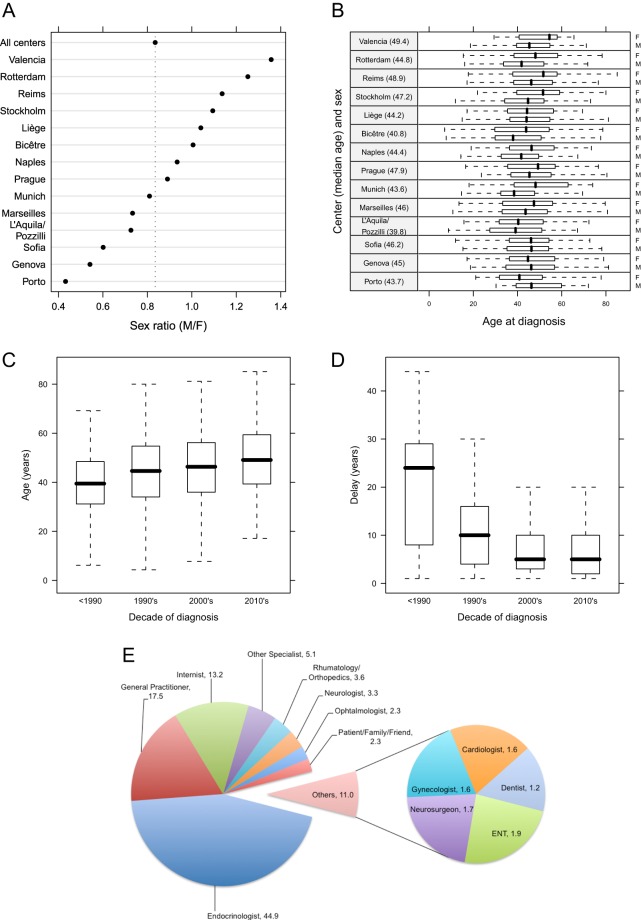
(A) Dot plot showing the sex ratio (M/F) and the number (*n*) of patients in the *LAS Database* and for individual centers. Centers were sorted based on the sex ratio, in decreasing order. (B) Median age of patients at diagnosis represented as separate boxplots for males and females. Centers were sorted based on the median age of diagnosis of all patients for each center (values in parenthesis). (C) Evolution of median age at diagnosis over time. (D) Estimated delay between the first symptoms of acromegaly as reported by patients and the diagnosis of acromegaly, and displayed by the decade of diagnosis. (E) Proportions of *LAS Database* patients diagnosed by different medical (generalist, specialist) or health care workers and non-medical individuals.

The study population consisted of 3173 patients with a diagnosis of acromegaly. There was a slight female predominance (*F* = 1729; 54.5%) across the total population, and this tended to decrease over time from 57.3% in those diagnosed before 1990 to 50.6% of those diagnosed after 2010. The male-to-female ratio (0.84) varied across the centers from 0.43 to 1.4 (Fig. 1A and B). A total of 468 cases underwent 777 genetic tests related to acromegaly; 73 patients had known genetic/inherited or syndromic features, 28 had an *AIP* gene mutation, 13 were from other *AIP-*negative familial isolated pituitary adenomas (FIPA) kindreds, 11 had McCune Albright syndrome, seven had multiple endocrine neoplasia type 1 (MEN1) and two had Carney complex. Five patients had acromegaly secondary to ectopic growth hormone-releasing hormone (GHRH) secreting tumors.

The median age of diagnosis was 45.2 years (IQR: 34.9–55.0 years) and was significantly younger in males (43.5 years (IQR: 34.2–53.1)) than that in females (46.4 years (IQR: 35.6–56.1); *P* < 0.001). The median age at first symptoms of acromegaly was 33.5 years (IQR: 23.6–44.5 years) overall and did not differ significantly between the sexes. The median delay in diagnosis was, however, significantly longer for females (10 years (IQR: 4.0–18.0)) as compared with males (8 years (IQR: 4.0–15.0); *P* = 0.015). The age at diagnosis increased over time in both sexes, with those in the most recent group (post-2010) being nearly 7 years older than the pre-1990 group (48.79 (39.3–58.9) vs 41.8 (32.5–52) *P* < 0.001; Fig. 1C). The median age at first symptoms of acromegaly (as recalled by the patient) also increased over time with patients diagnosed in the current decade being 17.1 years older than those diagnosed pre-1990 (41.7 (32.6–50.5) vs 24.6 (14–33.8); *P* < 0.001). Over time, however, acromegaly was associated with a shorter delay between first symptoms and diagnosis (Fig. 1D).

Acromegaly was most frequently diagnosed by endocrinologists (44.9%), general/family practitioners (17.5%) or internists (13.2%). Other diagnostic settings included rheumatologists/orthopedic specialists (3.6%), neurologists (3.3%) and ophthalmologists (2.3%), while in 2.3% of cases, the diagnosis was made by the patient themself or their family/friends (Fig. 1E). The most frequent signs/symptoms leading to presentation with acromegaly were changes in physical appearance, with 21.5% reporting dysmorphic features and 13.6% enlarged extremities. Other presenting signs included headache (7.5%), fatigue/asthenia (5.9%), sweating (2.0%) and sleep apnea (1.0%). In 8.4% of female patients, menstrual disturbances were among the symptoms leading to presentation with acromegaly.

### Radiological characteristics

**Figure 2:**
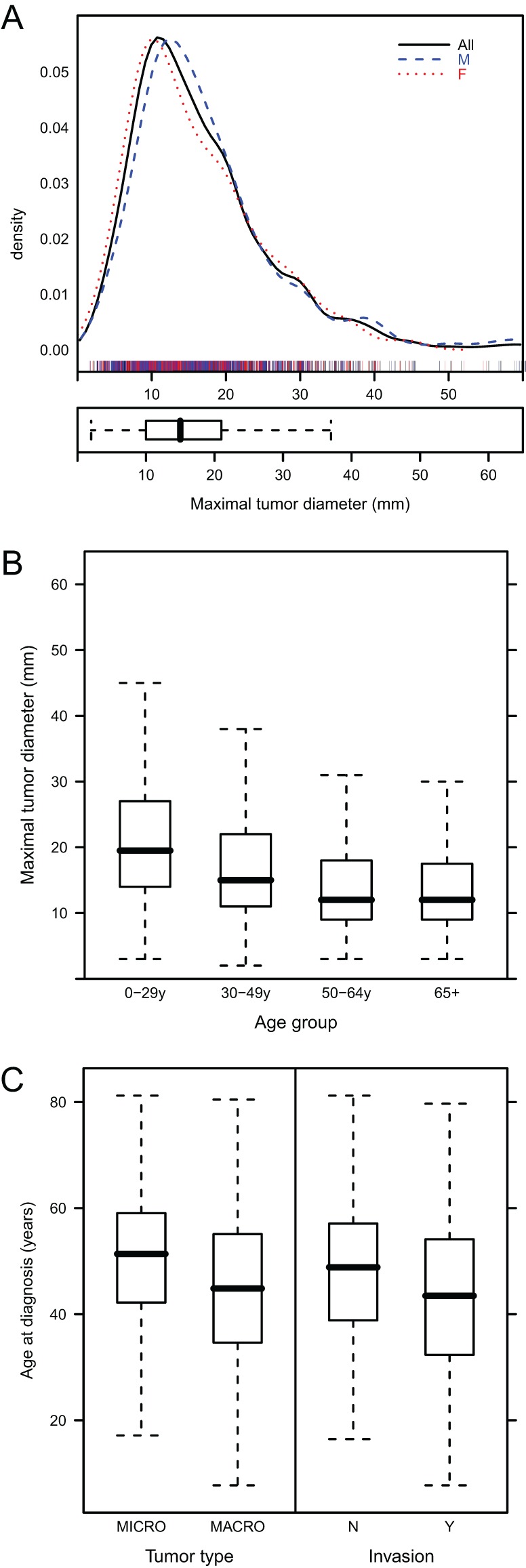
(A) Density plot and box plot representing the maximal diameter of tumor at diagnosis. Data for the whole population (black line), male (blue line) and female patients (red line) are shown. Individual patients are represented below the density plot (‘rug’). (B) Maximal tumor diameter in groups of patients based on the age at diagnosis. (C) Age of patients at diagnosis in those with micro/macro adenomas and in those with tumor invasion.

At diagnosis, pituitary imaging data were available in 2545 cases, of which 1691 had an MRI and 854 had a CT scan. The median tumor size at diagnosis was 15 mm (Fig. 2A) and 71.8% of cases had a macroadenoma. In 4.6% of cases, no pituitary tumor was visualized. Males had larger tumors at diagnosis than females (*P* < 0.001), while tumor size at diagnosis was inversely related to patient age (Fig. 2B). Hence, patients with macroadenomas were significantly younger (*P* < 0.001; Fig. 2C) and had more frequent cavernous sinus invasion at diagnosis (*P* < 0.001). The difference in tumor size between males and females was due to patients under 30 years of age at diagnosis (*P* = 0.002) as there was no significant difference between the sexes in tumor size in older patients (data not shown). In keeping with larger tumor size, younger patients had a higher rate of chiasmal compression at diagnosis, which was 23.0% in those aged <30 years but only 10.0% in those aged >65 years at diagnosis; this was present in both sexes (*P* < 0.001). The proportions of patients with micro/macroadenomas did not change over time. Invasion was present in 47.6% of tumors at baseline (Fig. 2C); there was no difference between the sexes and no change was seen in the percentage of cases with invasion over time.

### Hormonal profiles

**Figure 3:**
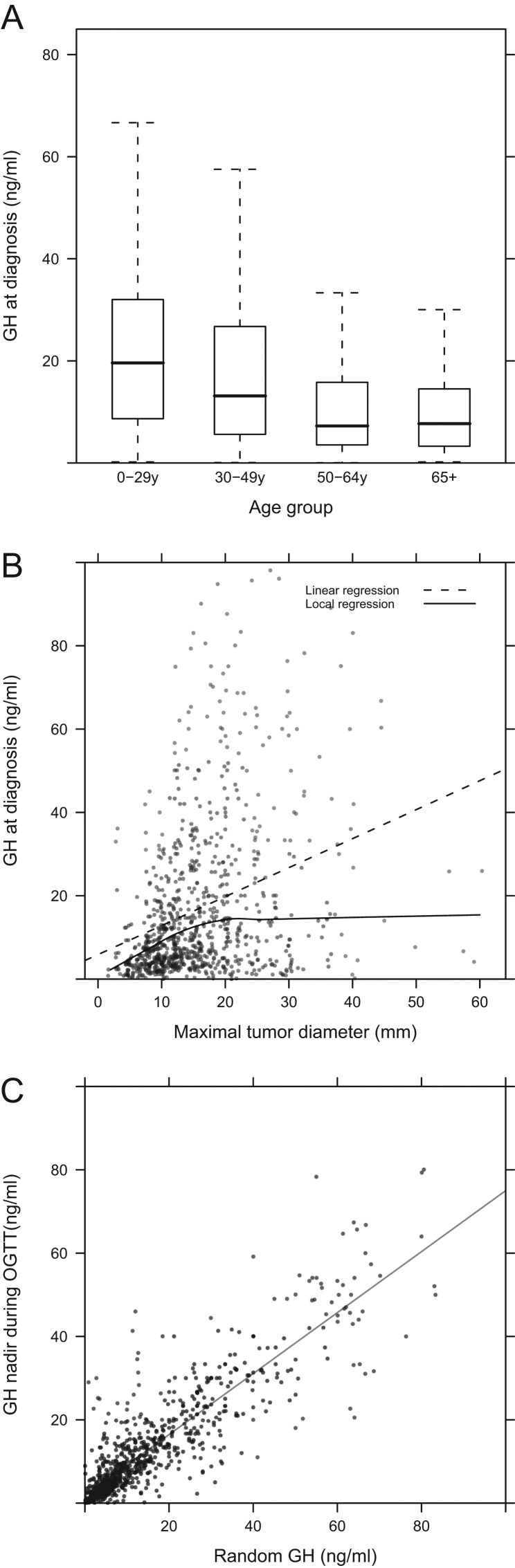
(A) GH levels in groups of patients based on the age at diagnosis. (B) Scatterplot of GH levels at diagnosis vs maximal tumor diameter. The dotted line is the linear regression between these two variables, whereas the continuous line is the result of a loess (locally weighted least squares regression) smoothing. The latter shows the lack of a correlation between tumor size and GH secretion for larger tumors. (C) Scatterplot and regression line between GH nadir concentration during OGTT vs random GH measurement.

At each age group studied, there was no difference between males and females in terms of GH level at diagnosis. GH levels at diagnosis were inversely related to patient age in both sexes (*P* < 0.001; Fig. 3A). A linear regression analysis between GH at diagnosis and maximal tumor diameter at diagnosis showed an increase of GH values with the size of tumor, but only up to a maximum tumor diameter of 20 mm; thereafter, no correlation with GH values existed (Fig. 3B). Random GH at diagnosis correlated closely with nadir GH levels during OGTT (*P* < 0.001, Fig. 3C). Over time, GH levels at diagnosis fell significantly; this was mainly driven by lower GH at diagnosis among females over time from pre-1990 to the current decade (*P* < 0001). As there was also a weak association between the date of diagnosis and the GH level, it cannot be excluded that changes in assay ranges could also contribute to this finding.

**Figure 4:**
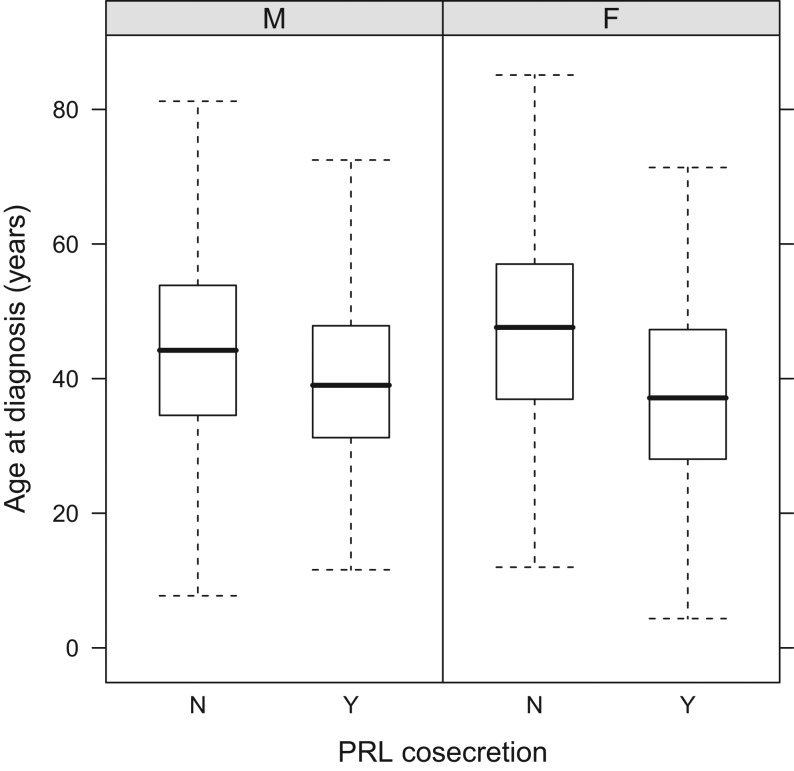
Age of male and female patients at diagnosis based on prolactin (PRL) co-secretion by the adenoma.

IGF-1 levels (%ULN) were higher at diagnosis among younger acromegaly patients; this difference was significant for the study population overall and male patients but not females (*P* < 0.001). IGF-1 (%ULN) also correlated with tumor size (*P* = 0.04). Prolactin co-secretion occurred in 10% of cases, while among surgically operated patients, mixed GH/PRL staining was described in 26.3% of tumors. Patients with prolactin co-secretion were significantly younger at diagnosis than other acromegaly patients (Fig. 4). Additionally, patients with GH and prolactin co-secretion had significantly larger tumors (*P* < 0.001) that were more likely to be invasive at diagnosis than other patients. Co-secretion of hormones other than prolactin was rarely seen at diagnosis (ACTH: 0.41%, TSH: 0.16%, gonadotropins: 0.13%).

## Comorbidities at diagnosis

### Metabolic system

**Figure 5:**
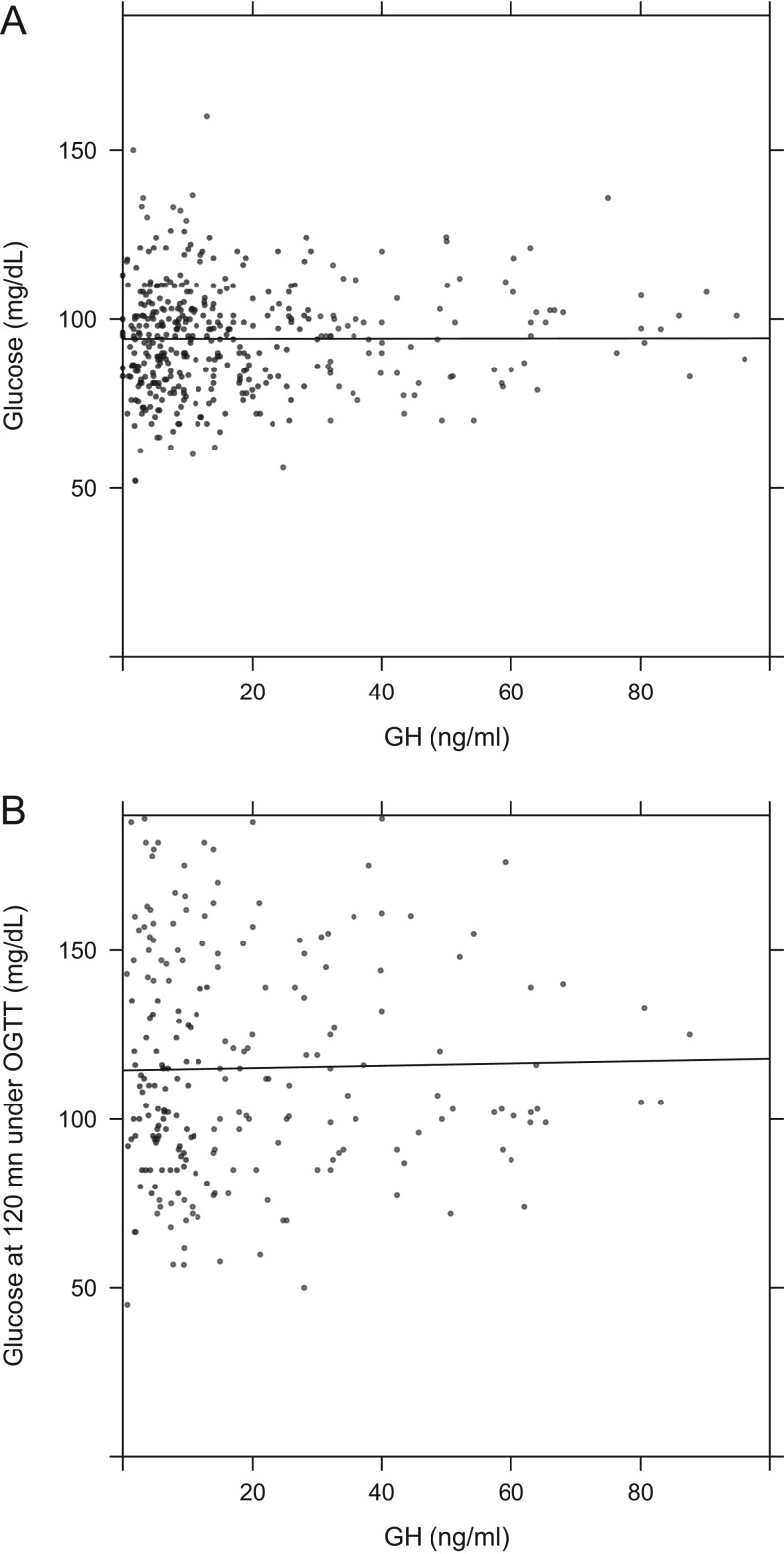
Scatter plots and regression lines of basal glucose (A) and glucose at 120 min during OGTT (B) vs GH levels in non-diabetic patients.

**Figure 6:**
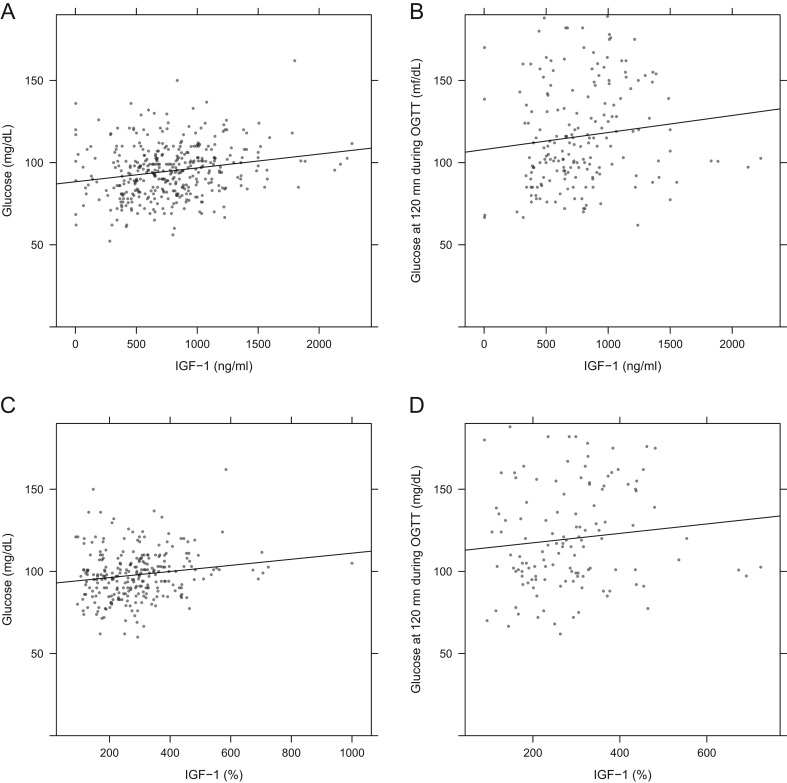
Scatter plots and regression lines of glucose vs IGF-1 levels in non-diabetic patients. (A) Basal glucose vs measured IGF-1. (B) Glucose at 120 min during OGTT vs measured IGF-1. (C) Basal glucose vs IGF-1 expressed as a percentage of the upper limit of normal (% ULN). (D) Glucose at 120 min during OGTT vs IGF-1 expressed as % of ULN.

At diagnosis of acromegaly, 24.5% of patients had type 2 diabetes, while three individuals had type 1 diabetes. In addition, when 120-min glucose values on OGTT were assessed, a further 24 patients not previously diagnosed with diabetes had glucose values >200 mg/dL at 120 min. Including all these patients, the prevalence of diabetes mellitus at diagnosis in acromegaly patients was 27.5%. In non-diabetic patients, glucose values (basal or at OGTT) did not correlate with GH levels (*P* = 0.19; Fig. 5A and B). Glucose levels did, however, correlate significantly with IGF-1 values when expressed in absolute terms (*P* < 0.01) and as %ULN (*P* = 0.038; Fig. 6A, B, C and D). The median total cholesterol level was 183.2 mg/dL (IQR: 134.0–216.2 mg/dL). Total cholesterol levels were higher in females than those in males at diagnosis: 189.0 mg/dL (IQR: 139.9–221.4) vs 178.0 mg/dL (IQR: 133.0–205.0). Males were nearly twice as likely to be current smokers as females at the time of diagnosis (22.1 vs 11.9%, respectively).

### Cardiovascular system

As cardiovascular disease is an important cause of morbidity/mortality in acromegaly, we were interested in determining the prevalence of important variables at diagnosis, in addition to diabetes and lipid profiles described previously. Among these, hypertension was the most frequent, occurring in 28.8% of patients overall at diagnosis, and this remained relatively constant across time of inclusion into the study. Cardiac hypertrophy was reported in 15.5% of patients at diagnosis. Other important cardiovascular morbidities were less frequent at diagnosis: stroke (4.5%), arrhythmia (3.6%), ischemic heart disease (3.5%), myocardial infarction (3.0%) and heart failure (1.6%). Patients with hypertension, cardiac hypertrophy, cardiac failure, ischemic heart disease and arrhythmia at diagnosis were all significantly older at diagnosis than those without these cardiovascular comorbidities (*P* < 0.001). Sleep apnea syndrome had been diagnosed in 25.5% of the cohort. In centers where polysomnography was systematically performed, sleep apnea syndrome was detected in 69.0% of tested subjects.

Red blood cell counts (analyzed separately for males and females) did not show any correlation with random GH or GH nadir during OGTT (*P* = 0.46), but RBC count increased with absolute IGF-1 values (*P* = 0.046) and %ULN values (*P* < 0.001). Similarly, hemoglobin concentration was not correlated with GH levels but was positively correlated with absolute IGF-1 values (*P* = 0.017) and IGF1 %ULN (*P* < 0.001).

### Other comorbidities

Overall, 34.0% of patients had a thyroid nodule or goiter reported at diagnosis. There was no relationship between other demographic or hormonal factors and the presence of thyroid nodules. At diagnosis, 13% of patients who had a colonoscopy (*n* = 820) had colonic polyps identified. There was no difference in GH and IGF-1 levels between patients with and without polyps. Four patients had been diagnosed with colorectal cancer at diagnosis. In total, 64 patients had a diagnosis of cancer, the most common of which were breast (*n* = 16), thyroid (*n* = 11) and skin (*n* = 10). At diagnosis, 12.3% of patients had been diagnosed with osteoporosis. A hip fracture had occurred by the time of diagnosis in 4.4% of acromegaly patients, whereas 4.3% had suffered a vertebral fracture and 0.6% a wrist fracture. There was only a significant relationship between age at diagnosis and the presence of any fracture in female patients (*P* = 0.012).

## Discussion

Acromegaly is a rare endocrine disorder that is due to chronic GH hypersecretion, usually from a pituitary adenoma. It is usually diagnosed and managed in expert referral centers, but due to its rarity even pituitary specialists might see only a couple of hundred cases over their full careers. One way to improve our understanding of rare disorders is by pooling data from many centers using patient registries. In acromegaly, this has been done extensively on a regional and national basis in Europe and Mexico (Jenkins *et al.* 1995, Mestron *et al.* 2004, Reincke *et al.* 2006, Bex *et al.* 2007, Portocarrero-Ortiz *et al.* 2016, Maione *et al.* 2017). Commercial entities support clinical databases to detect and report on the safety of medical therapies, such as, the pegvisomant ACROSTUDY program (van der Lely *et al.* 2012, Freda *et al.* 2015, Bernabeu *et al.* 2016). The data obtained from these registries have stimulated ideas on aspects of morbidity, hormonal control and medical treatment patterns that have later been proven in independent clinical trials. National registries do have limitations in terms of patient numbers and the applicability of data to treatment norms in other countries. International acromegaly databases with a common underlying data capture methodology have been long called for (Stewart 2004).

The *LAS Database* was originally developed and validated as a single-center study tool (Petrossians *et al.* 2012), and thereafter, was expanded across multiple European centers in the current study; it has been used successfully to facilitate analyses of disease characteristics and treatment responses in various centers (Theodoropoulou *et al.* 2009, Franck *et al.* 2017). The *LAS Database* provides some specific advantages in that it is not limited to a national dataset nor does it deal with patients managed with a single treatment modality. The programming of the *LAS Database* is a relational database that permits integrated statistical analyses of independent variables, which is a challenge for other registry-based listing. The *LAS Database* variables (nearly 150 in total)were chosen based on extensive input from acromegaly specialists in order to permit clinically relevant questions and changes in criteria over time to be addressed with robust statistical methods.

In the cohort, there was a small female predominance overall (54.5%), which is in keeping with results from other national centers in Europe and elsewhere (Sesmilo *et al.* 2012, Portocarrero-Ortiz *et al.* 2016, Lesén *et al.* 2017, Maione *et al.* 2017). Over time, though, the sex prevalence changed, such that those patients diagnosed post-2010 were nearly evenly balanced (M:F 49.4%: 50.6%). Acromegaly usually has an occult onset and a long period of symptoms can occur before a diagnosis is made. In a two-center study in the United States, Reid and coworkers suggested that delayed diagnosis contributed to acromegaly patients presenting with similar disease characteristics over the period 1981–2006 (Reid *et al.* 2010). In the *LAS Database*, first symptoms were seen in the mid-30s in both sexes. However, it took significantly longer (2 years) for females to achieve a diagnosis than males, which is clinically relevant and indicates improved awareness of acromegaly in women is needed. As the delay between first symptoms and diagnosis decreased over the course of the study, this suggests that the efficiency of referral and diagnosis is improving. This may be due to much wider access to MRI and other specialist techniques and better emphasis on concentrating pituitary expertise in regional referral centers; improved awareness of acromegaly may itself play a part in decreasing the delay before diagnosis. It is interesting to note that the age at diagnosis in the cohort overall increased by nearly seven years from 1990 to the current decade. It has long been noted that older patients with acromegaly can have milder disease features and hormonal abnormalities (van der Lely *et al.* 1992). More recently, it has been noted that a group of patients with ‘normal’ GH and elevated IGF-1 exists, that are older and have smaller tumors than acromegaly patients with typically raised GH and IGF-1 parameters (Dimaraki *et al.* 2002, Butz *et al.* 2016). It may be that the wider access to MRI and greater awareness noted above is also leading to increased pick-up of a milder phenotype of acromegaly in an older population. In support of this, hormonal data from the current cohort show a fall in GH at diagnosis over time, due mainly to female acromegaly patients. The correlations between patient age, tumor size and GH secretion suggest an apparent triangular relation among these three variables. The later the age at diagnosis, the smaller the tumors and the lower the diagnostic GH values; the reverse situation was also true. This raises different possible interpretations. Is milder disease simply being overlooked in younger patients or are older patients more sensitive to small increases in GH secretion? It is more likely, however, that acromegaly is heterogeneous, and there are distinct phenotypes that can be identified. A number of pathological features might explain this difference, including genetic causes, such as *AIP* mutations that predominately affect younger males (Daly *et al.* 2010). Over representation of *AIP-*mutated cases among younger subgroups of the current cohort could have influenced the tumor size characteristics. As only a minority of patients underwent tumoral or germline genotyping, this hypothesis remains speculative. GH values at diagnosis decreased with patient age and increased with tumor size, although this later linear relation was not present for tumors measuring more than 20 mm in diameter. This may be explained by tumoral necrosis in bigger tumors or by two different populations of tumors with the bigger tumors being aggressive tumors secreting relatively low levels of GH that appear as hyper-intense lesions on T2-weighted MRI sequences (Potorac *et al.* 2015, 2016). Further studies comparing T2 imaging signal, histologic features and tumoral secretion may shed more light on this observation.

Improvements in diagnosis of acromegaly can come from greater awareness among those who first see the patient. In the *LAS Database* cohort, the initial diagnosis of acromegaly was made by an endocrinologist in nearly 45% of cases. As shown in Fig. 1, the variety of non-endocrine specialists that make acromegaly diagnoses is quite marked. Given the range of potential signs/symptoms and the specific problems caused by a pituitary adenoma, it is crucial that awareness of pituitary tumors continues to be widened across medical specialties and related groups (Surchi *et al.* 2017). Delays in diagnosis in patients that attend with multiple symptoms of acromegaly still occur as illustrated clearly by De and Foucault, leading to unnecessary exposure of excessive GH/IGF-1 (De & Foucault 2014). Interestingly, in the age of widespread Internet searching related to medicine, as many people or friends/family diagnosed himself or herself with acromegaly as did ophthalmologists. Improved understanding of the pattern of signs and symptoms suggestive of acromegaly is still needed among both the health care sector and the general public.

Studies in acromegaly routinely use random GH measurements, whereas the nadir of GH during OGTT is considered as the ‘gold standard’ of GH assessment. In this cohort of >3100 patients, a linear regression between nadir GH and random GH showed a good correlation between these two measures suggesting that using random measurement of GH is a clinically valid practice, as suggested by others (Bajuk Studen & Barkan 2008). Despite extensive clinical research, the question still arises as to which hormonal measurement, GH or IGF-1 (or both), is the best representation of the activity of acromegaly. Indeed in clinical practice, patients with high levels of GH and comparatively low (albeit elevated) levels of IGF-1 are seen, contrasting with other patients with slightly increased or normal levels of GH but markedly elevated levels of IGF-1. Which of these patients should be considered as being the most exposed to active acromegaly? One pointer may come from comparing other biological markers like glucose. Detailed study of acromegaly patients with diabetes is limited since these patients are already receiving treatment, and they may show different compliance toward their diet and therapy. Therefore, we assessed the effect of hormonal secretion in non-diabetic patients. Glucose levels in acromegaly patients increased with rising levels of IGF-1, whereas no correlation was found with GH. Although GH induces insulin resistance and raises glucose, in the clinical setting, IGF-1 may represent a better marker of the metabolic burden of acromegaly; this point is echoed in other national cohort analyses (Alexopoulou *et al.* 2008).

Acromegaly is associated with increased mortality when hormonal levels are not controlled (Dekkers *et al.* 2008). The presence of important comorbidities contributes to this and the range of pathologies seen in acromegaly patients is extensive (Pivonello *et al.* 2017). The actual contribution of the different major classes of comorbidity to disease burden and death in acromegaly is not clear. Traditionally, cardiovascular disease, respiratory disease and cancer have been the main causes of increased mortality in acromegaly. With respect to cardiovascular and metabolic risk factors in the current cohort, we confirm that diabetes is a common problem in acromegaly, affecting more than a quarter of patients at diagnosis, in keeping with other studies (Hannon *et al.* 2017). Hypertension was also frequent, being present in about 29% at diagnosis. Structural heart disease is an important component of acromegaly, and already 15.5% of patients had hypertrophy at diagnosis. We noted that sleep apnea syndrome, a classical acromegaly feature, that itself has a negative impact on cardiorespiratory morbidity is seen in a quarter of acromegaly patients at diagnosis. This figure is likely to be an underestimate, as with strict polysomnography, the rate of obstructive sleep apnea syndrome in acromegaly can be as high as nearly 70% (Attal & Chanson 2010). Acromegaly patients are not screened uniformly at diagnosis for sleep apnea or other associated problems, so the true prevalence rates of different comorbidities are uncertain. An important factor to consider is the effect of age on cardiovascular comorbidities, as we noted that patients with hypertension, cardiac hypertrophy and heart failure at diagnosis were significantly older at diagnosis (6–13 years) than those without cardiovascular complications. This raises the question as to what role acromegaly plays in the cardiovascular health of the aging patient? This is particularly of relevance as the current study has shown that more aged patients with acromegaly are being diagnosed. In this situation, it becomes difficult to attribute a causative role for GH hypersecretion to cardiac morbidities in acromegaly, and as patients age, the presence of acromegaly may simply represent one of the many contributory risk factors.

In the case of colonoscopy that is recommended for surveillance of acromegaly patients, this was performed in 820 patients at diagnosis. While incomplete with respect to the total cohort size, it is still one of the largest datasets on colonic findings at diagnosis in acromegaly; 13% of patients had polyps but only four cases of colorectal cancer were already present at diagnosis. Indeed, the rate of recorded cancer cases either overall or by specific types (e.g. breast cancer in women) does not appear as being markedly elevated in the *LAS Database* patients in relation to general European populations (Lutz *et al.* 2003). The prevalence of thyroid nodules was high in acromegaly at diagnosis; and 11 cases of thyroid cancer were identified at that time. The prevalence of thyroid nodules was probably an underestimation as ultrasound examinations were not performed routinely at diagnosis of acromegaly. There were some interesting findings regarding emerging comorbidities. Red blood cell count and hemoglobin concentrations were also raised in acromegaly, and we confirmed that these increased with IGF-1 levels but not GH. Again, this suggests that IGF-1 levels may be a better representation of the activity of acromegaly overall. The role of excessive GH-IGF-1 hypersecretion on erythropoiesis in acromegaly is a recognized but relatively neglected subject (Grellier *et al.* 1996, Teramoto & Ouchi 1997, Zoppoli *et al.* 2011); however, in pediatric and adult GH deficiency, it is well established that GH replacement can lead to increased red blood cell measure and correction of anemia (Christ *et al.* 1997, Valerio *et al.* 1997, Bergamaschi *et al.* 2006, Esposito *et al.* 2016). The role of increased red cell counts and potentially other hematological measures in relation to respiratory pathology (e.g. sleep apnea syndrome), cardiovascular disease and outcomes is a potentially valuable avenue of future research.

The *LAS Database* is the first international relational database used to study acromegaly following a standard methodological design. This first report of >3100 enrolled patients at diagnosis shows that the clinical and hormonal characteristics of acromegaly are evolving over time. While acromegaly affects slightly more females than males, female patients have a significantly longer delay before diagnosis; this may be due in part to males having larger tumors than females and these occur at a younger age. The age at first symptoms and at diagnosis of acromegaly is rising over time, indicating that improvements in diagnostic measures are detecting a greater proportion of older patients. In keeping with this, the *LAS Database* cohort also shows a triangular relationship between age, tumor size and GH secretion, with older patients having smaller tumors and lower GH secretion. Cardiometabolic comorbidities of acromegaly were frequently present at diagnosis, such as diabetes mellitus (29.6%), hypertension (28.8%), while cardiac hypertrophy was seen in 15.5%. Thyroid nodules (34.0%), sleep apnea syndrome (25.5%) and colonic polyps (13%) were also frequent but detailed specific screening for these was less consistent at diagnosis. The *LAS Database* provides a standardized platform for combining large datasets across multiple centers internationally and forthcoming analyses will address important aspects of treatment responses and outcomes in acromegaly.

## Declaration of interest

Patrick Petrossians has undertaken consulting and has received travel grants from Novartis, Ipsen and Pfizer. Adrian F Daly holds stock in Amryt Pharma. Annamaria Colao has been principal investigator of research studies from Novartis, Ipsen, Pfizer and Lilly, has received research grants from Ferring, Lilly, Ipsen, Merck-Serono, Novartis, Novo-Nordisk and Pfizer, has been a consultant for Novartis, Ipsen and Pfizer and has received fees and honoraria from Ipsen, Novartis and Pfizer. Renata S Auriemma has been a consultant for Novartis and has received fees and honoraria from Novartis. Sebastian Neggers has received research grants from Ipsen, Pfizer and Novartis and has been a consultant for Pfizer and Ipsen. Vaclav Hana has received speaker fees and has served on Advisory Boards for Pfizer, Novartis and Ipsen. Albert Beckers has received research grants from Ipsen, Pfizer and Novartis and has served on Advisory Boards for Ipsen.

## Funding

This study was supported by an unrestricted educational grant from Ipsen. The study funder had no role in the collection of data, had no access to the data and had no involvement in the writing of this manuscript.
